# Study on high-CO_2_ tolerant *Dunaliella salina* and its mechanism *via* transcriptomic analysis

**DOI:** 10.3389/fbioe.2022.1086357

**Published:** 2022-12-01

**Authors:** Bo Huang, Gaopin Qu, Yulong He, Jinli Zhang, Jianhua Fan, Tao Tang

**Affiliations:** ^1^ CAS Key Lab of Low-Carbon Conversion Science and Engineering, Shanghai Advanced Research Institute, Chinese Academy of Sciences, Shanghai, China; ^2^ State Key Laboratory of Bioreactor Engineering, East China University of Science and Technology, Shanghai, China

**Keywords:** *D. salina*, high-CO2 tolerance, antioxidant system, transcriptomic analysis, high-salt tolerance

## Abstract

Microalgae has been regarded as a promising method for reducing CO_2_ emission. High CO_2_ concentration generally inhibits algal growth, and previous studies have mostly focused on breeding freshwater algae with high CO_2_ tolerance. In this study, one marine algal strain *Dunaliella salina* (*D. salina*) was grown under 0.03%-30 % CO_2_ and 3% NaCl conditions, and was evaluated to determine its potential for CO_2_ assimilation. The results showed that *D. salina* could tolerate 30% CO_2_ *,* and its maximum biomass concentration could reach 1.13 g·L^−1^ after 8 days incubation, which was 1.85 times higher than that of incubation in air (0.03%). The phenomenon of high-CO_2_ tolerance in *D. salina* culture was discussed basing on transcriptome analysis. The results showed that *D. salina* was subjected to oxidative stress under 30% CO_2_ conditions, and the majority genes involving in antioxidant system, such as SOD, CAT, and APX genes were up-regulated to scavenge ROS. In addition, most of the key enzyme genes related to photosynthesis, carbon fixation and metabolism were up-regulated, which are consistent with the higher physiological and biochemical values for *D. salina* incubation under 30% CO_2_ *.*

## 1 Introduction

Carbon dioxide (CO_2_) accounts for 68% of greenhouse gas emission as a result of human activity (Zhou L et al., 2017). Approximately 33.4 Gt of CO_2_ is emitted to the Earth’s atmosphere each year, and approximately 40% of it is generated from fossil fuel power plants (Singh et al., 2019). It is extremely important to develop technologies for CO_2_ capture, utilization and storage (CCUS) that can be conducted at low energy consumption and cost.

Compared with the chemical absorption and geologic sequestration of CO_2_, biological CO_2_ capture using microalgae has been regarded as a promising new method for reducing CO_2_ emission (Wang H et al., 2018). The CO_2_ concentration of flue gas in the power plants is usually 10%–20%. However, most of microalgae grow only at low CO_2_ concentrations level, and would be inhibited when CO_2_ concentration level was higher than 5% (Thomas et al., 2016). Only a few microalgal species have been reported to tolerate extremely high CO_2_ level up to 70% and even 100% (Ota et al., 2009; Ho et al., 2010; Bhakta et al., 2015). However, most of these promising microalgal species with high CO_2_ tolerance are freshwater species. Generally, huge amounts of water are needed in microalgal cultivation. Freshwater resources are limited in some countries and regions, and water recycling increases operational costs and risks (Rodolfi et al., 2003; Depraetere et al., 2015; Lu et al., 2019; Lu et al., 2020). To avoid competition with freshwater resources, saline water and wastewater may be possible choices for microalgae cultivation. However, the heavy metals and other pollutes in industrial wastewater led to safety risk in the applications of microalgal biomass (Sharma et al., 2022; You et al., 2022). The use of saline water to cultivate microalgae is an ideal solution for microalgae production, which can not only reduce the production costs, but also reduce the pressure of freshwater consumption (Ishika et al., 2017). Therefore, it is necessary to identify and develop certain marine microalgal strains that can grow under saline water and high CO_2_ concentration. Only a few studies have investigated the effect of CO_2_ concentrations on the growth of marine microalgal species, such as *Nannochloropsis oculate* (Chiu et al., 2009), *Thalassiosira weissflogii* (Ishida et al., 2000), *Chaetoceros muelleri* (Wang et al., 2014), *P*. *glacialis and A*. *longicornis* (Artamonova et al., 2017) and *Phormidium valderianum* (Dineshbabu et al., 2020). Chiu et al. (2009) investigated the effects of CO_2_ concentration (0.03%–15%) on the biomass production of *Nannochloropsis oculata* NCTU-3. The highest biomass concentration (1.28gL^−1^) was obtained under 2% CO_2_. 5%–15% CO_2_ were harmful to microalgal cells and inhibited their growth. Wang et al. (2014) investigated marine diatom *Chaetoceros muelleri* in response to different CO_2_ levels (0.03%–30%). It was found that *Chaetoceros muelleri* showed maximum biomass concentration (1.06 g L^−1^) under 10% CO_2_. However, higher CO_2_ concentrations led to negative effect on microalgal growth, and the biomass concentrations sharply reduced to 0.59 and 0.31 g L^−1^ under 20% and 30% CO_2_, respectively. Ishida et al. (2000) reported one high CO_2_-tolerant microalgal strain isolated and identified as *Thalassiosira weissflogii* H1. There was no significant difference between the growth rates and maximum growth yields of this diatom under bubbling air, 5% CO_2_ and 10% CO_2_, but the growth rate and maximum growth yield under 20% CO_2_ markedly decreased. Wang S et al. (2018) invested two oil-rich microalgal strains *Isochrysis galbana* and *Nannochloropsis sp*.in response to CO_2_ aeration. The results showed that maximum biomass concentrations of *Isochrysis galbana* and *Nannochloropsis sp.* were around 0.75 g L^−1^ under 10% CO_2_, and reduced to around 0.55 g L^−1^ under 15% CO_2_. Dineshbabu et al. (2020) studied the effect of CO_2_ concentrations to the growth of marine cyanobacterium *Phormidium valderianum*. The maximum biomass productivity was 83.33 mg L^−1^ d^−1^ was recorded under 3% CO_2_, and decreased to 51 mg L d^−1^ under 15% CO_2_, which meant higher CO_2_ concentration inhibited microalgal growth. The above results indicated high CO_2_ concentrations showed significant inhibition to the microalgal growth under high salt conditions. Therefore, it is urgent to develop microalgal strain with high-CO_2_ tolerance and high-salt tolerance.

In this study, we investigated the effect of CO_2_ concentrations on the growth of one marine microalgal strain *D. salina* under high-salt conditions. Transcriptomic analysis was used to investigate the factors affecting microalgal tolerance for high CO_2_ concentration at a genetic level. These findings extend the knowledge which the key metabolic and biological pathways function in response to extremely high CO_2_ in marine microalgal strain.

## 2 Materials and methods

### 2.1 Strain and growth conditions

one marine microalgal strain *D. salina* (GY-H13) was purchased from Shanghai Guangyu Biological Technology Co., LTD, and maintained in petri dishes using BG11 solid medium containing 3% NaCl. *D. salina* cells were successively transferred from petri dishes to 250 ml flasks, and then cultivated in 400 ml bubble column photobioreactors (PBR, working volume 300 ml) with 1% CO_2_ under 110 μmol m^−2^ s^−1^ and 25°C conditions. The cells of *D. salina* were harvested during their logarithmic growth phase by centrifugation, and then the harvested cells were resuspended into BG11 medium containing 3% NaCl with the required biomass density and used in the following experiments.

In order to investigate the effect of CO_2_ concentration on the growth of *D. salina*, the microalgal cells were cultured at five CO_2_ levels, air (0.03%CO_2_), 1%CO_2_, 10%CO_2_, 20%CO_2_, and 30%CO_2_. The culture was incubated in 400 ml PBR (working volume 300 ml) under 110 μmol m^−2^ s^−1^ and 25°C conditions. The concentration of CO_2_ (v/v) was manipulated by adjusting the flow rates of pure CO_2_ and air with gas mass flow controllers, respectively. The mixed gases were filtered (0.22 µm) and then transferred into the bottom of PBR through one slender glass tube (inner diameter 0.25 cm) with an aeration rate of 0.2 L min^−1^. The initial cell density of the cultures was maintained at 0.1 g L^−1^. The temperature was maintained using constant temperature water bath.

### 2.2 Growth and chemical composition measurements

The cells of *D. salina* were cultivated for 8 days at 0.03%–30% CO_2_. Samples were taken daily from the PBR to estimate microalgal growth and the pH of the culture medium. Generally, 12 ml of culture medium was collected in a clean glass tube, and the pH of the sample was immediately measured using a Five Easy pH meter (METTLER TOLEDO). The maximum quantum yield of photosystem Ⅱ was determined using 2 ml of the sample. The F_v_/F_m_ ratio was measured using a fluorescence monitoring system (FMS2, Lufthansa Scientific Instruments Co., Ltd., United Kingdom) after the sample has been stored in dark conditions for 30 min (García-Cañedo et al., 2016). Of the remaining sample 10 ml was filtered using a pre-dried and pre-weighed cellulose membrane (0.45 µm pore size), washed with deionized water, dried for 24 h at 105°C, cooled in a desiccator and then weighed again to determine uncorrected dry algae biomass. The dry weight of the blank filter was subtracted from that of the loaded filter to obtain the corrected algae dry cell weight. At the end of the microalgal cultivation period (8 days), microalgal samples were harvested to test the contents of Chlorophyll and Carotenoid content. The pigments were extracted and calculated as described in Pruvost et al. (2011). The absorbances at 480, 652, 665, and 750 nm were measured by HACH DR2800 in a glass cell with a path length of 1 cm.

### 2.3 Transcriptome functional annotation and differential expression analysis

Transcriptomic analysis was used to investigate and test potential CO_2_ tolerance mechanisms at a genetic level. *D. salina* cells cultivated under air (0.03%), 1% and 30% CO_2_ were collected on day 1 and day 2, and denoted as C (0.03%) D1, C (1%) D1 and C (30%) D1; C (0.03%) D2, C (1%) D2 and C (30%) D2, respectively. Each sample was determined for two replicates. The sample treatment, transcriptomic determination, gene annotation and the related bioinformatics analysis have been described in previous studies (Zhou W et al., 2017; Cheng et al., 2019). NCBI non-redundant protein (NR) database classification of transcriptome sequences using DIAMOND software. Swiss-Prot database classification of transcriptome sequences using DIAMOND software. Protein family (Pfam) database classification of transcriptome sequences using HMMER3 software. Protein direct homology cluster (COG) database classification of transcriptome sequences using DIAMOND software. Gene ontology (GO) classification of transcriptome sequences using BLAST2GO software. Kyoto Encyclopedia of Genes and Genomes (KEGG) classification of transcriptome sequences using KOBAS software. Expression levels of genes and transcripts were quantified separately using the expression quantification software RESM, quantified as FPKM (fragments per kilobase exon model per million mapped reads), FPKM takes into account the effect of gene length and sequencing volume differences on the calculation of gene expression by first homogenizing the sequencing volume and then the gene length to allow visual comparison of expression. In order to control the probability or frequency of errors in the overall inferred results, the *p*-values obtained from the statistical tests are corrected using BH (FDR correction with Benjamini & Hochberg) for multiple testing, and the corrected *p*-values are known as p-adjust. Differential folds FC (Fold change) for different CO_2_ concentration treatments were calculated based on the FPKM values of gene expression using RSEM software for differential analysis. Genes meeting the screening criteria of |log_2_ FC|≥1 & p-adjust <0.05 were considered as differentially expressed genes (DEGs). GO and KEGG enrichment analyses were performed on genes in the gene set using Goatools. Raw data was deposited at the NCBI Sequence Read Archive (SRA) with BioProject record PRJNA901516.

## 3 Results and discussion

### 3.1 Effect of different CO_2_ treatments on physiological indicators of *D. salina*

**FIGURE 1:**
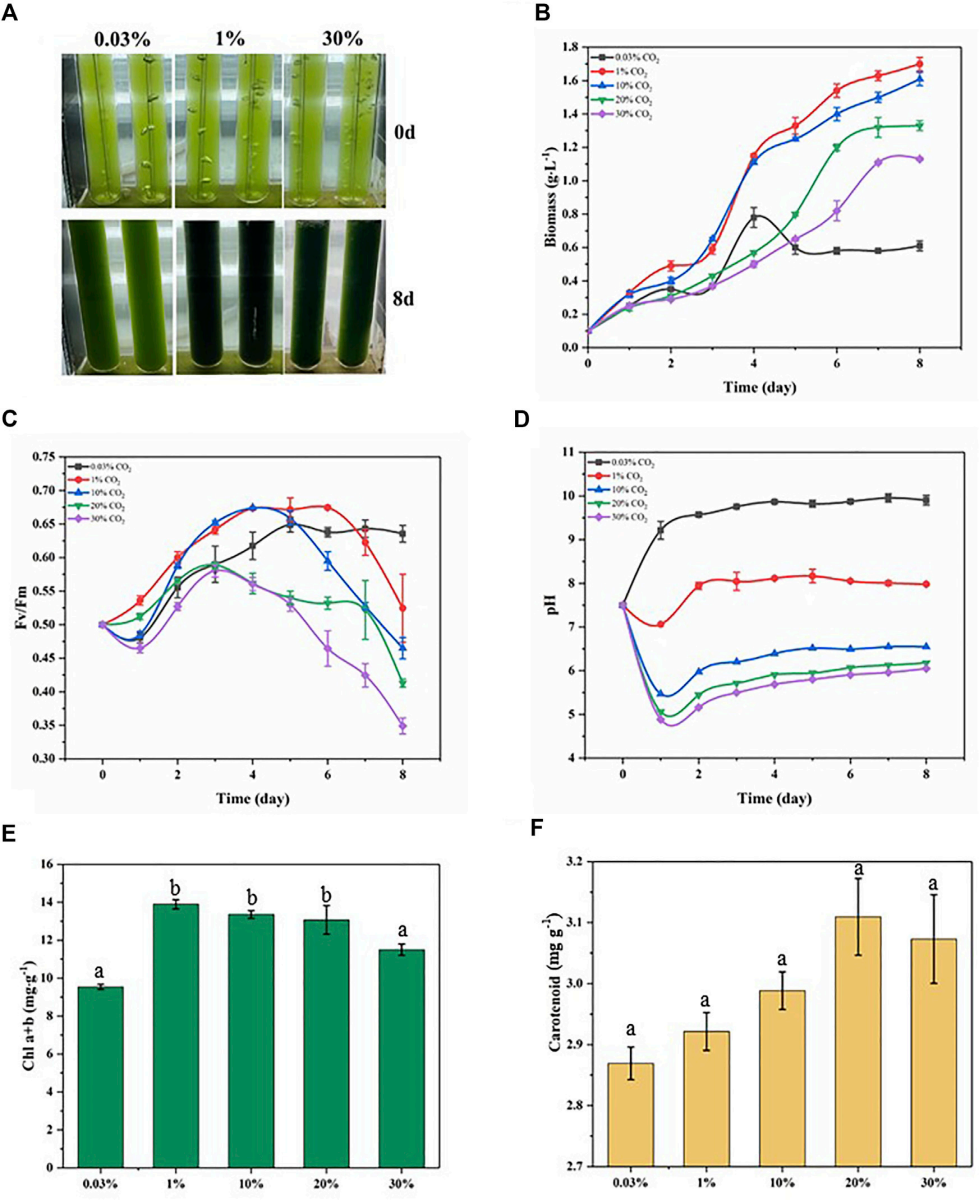
Physiological parameters related to growth status in microalgae exposed to different CO_2_ conditions within 8 days treatments. **(A)** Realistic image; **(B)** Biomass; **(C)** the maximum quantum efficiency of photosystem II (F_v_/F_m_); **(D)** pH; **(E)** Chlorophyll content; **(F)** Carotenoid content.

As shown in Figure 1B, the growth of *D. salina* was significantly influenced by the CO_2_ level. The biomass of microalgal cells was 0.61 g L^−1^ after 8 days of incubation in air (0.03%CO_2_). The cell biomass of the 1% CO_2_ group grew rapidly, reaching 1.7 g·L^−1^ on day 8, which was 2.79 times higher than that of incubation in air. Further increasing CO_2_ concentrations resulted in the lower biomass concentrations under 10%–30% CO_2_. However, the cell biomass of 30% CO_2_ group also reached 1.13 g·L^−1^, which was 1.85 times higher than that of incubation in air. The effect of different CO_2_ concentration treatments on the growth of *D. salina* is further evidenced by the picture of *D. salina* growth*.* (Figure 1A). Under the similar experimental conditions, the growth parameters of *D. salina* used in this study are higher than previous marine microalgal strain. (Ishida et al., 2000; Chiu et al., 2009), *Chaetoceros muelleri* (Wang et al., 2014), *P. glacialis* and *A. longicornis* (Artamonova et al., 2017) and *Phormidium valderianum* (Dineshbabu et al., 2020). These results suggest that *D. salina* can tolerate a high CO_2_ concentration environment.

Chlorophyll fluorescence parameters have been gradually applied in the study of the effects of environmental stress on cell photosynthesis (Bhagooli et al., 2021). Among the many parameters, the parameter F_v_/F_m_ has little change under non-stress conditions and decreases significantly under stress conditions. It is suitable parameter to reflect the influence of environment on the growth of microalgae. Under 0.03% CO_2_ conditions, the F_v_/F_m_ ratio decreased to 0.48 on day 1, then gradually increased to 0.65 on day 5, and finally remained around this value until day 8. Under 1% CO_2_ conditions, the F_v_/F_m_ ratio increased to 0.54 on day 1, reached a maximum of 0.67 on day 6, and then decreased to 0.53 on day 8. Under 30% CO_2_ conditions, the F_v_/F_m_ ratio decreased to 0.47 on day 1, then gradually increased to 0.58 on day 3, and finally gradually decreased to 0.35 on day 8 (Figure 1C). The variation of F_v_/F_m_ ratios under different CO_2_ conditions indicated that the 1% CO_2_ treatment condition was more suitable for *D. salina* growth. In contrast, the changes of F_v_/F_m_ ratios at 30% CO_2_ showed a reduced photochemical efficiency through damage to PSII under higher CO_2_ stress. These results show consistency with the growth of *D. salina*.

The pH of the culture medium affects the permeability of the cell membrane of microalgae cells, the uptake and utilization of ions in the culture medium, the utilization of CO_2_, and may alter the nutrient metabolism of microalgal cells (Barati et al., 2021). The starting pH of the culture medium was 7.8. Under 0.03% CO_2_ conditions, the pH increased to 9.2 on day 1, then continued to increase and remained around 9.6. Under 1% CO_2_ conditions, the pH dropped to 7.06 on day 1, then increased to 7.94 on day 2, and finally remained around 8.05. Under 30% CO_2_ conditions, the pH dropped to 4.89 on day 1, then increased to 5.5 on day 3, and finally remained around 5.9 (Figure 1D). It can be seen that a pH-shift occurred for all cultures resulting in more alkaline conditions over time, and the pH level decreased with increasing CO_2_ concentration. Generally, the pH of the culture medium was dictated by processes of acidification resulting from CO_2_ dissolution and alkalization due to microalgal photosynthesis removing dissolved CO_2_. The pH of the culture medium increased with time because of photosynthesis which elevated pH after algae acclimation in the culture medium. Alkalization of the culture medium is thought to compensate for the acidification effect of high CO_2_ concentration (Solovchenko and Khozin-Goldberg, 2013). At higher CO_2_ concentrations (10–30%), the combination of greater acidification and diminished photosynthesis resulted in the lower pH range.

Chloroplasts are important organelles in most photosynthetic microalgae, and chloroplasts also serve as sensors of the external environment (Zhang et al., 2020). Chlorophyll content is an important indicator of the photosynthetic intensity of microalgal cells, and the adaptation of microalgal cells to different CO_2_ concentration conditions can be detected by chlorophyll content (Kumari et al., 2021). We determined the chlorophyll contents of *D. salina* cells after 8 days of incubation under different CO_2_ concentrations conditions. As shown in Figure 1E, the maximum chlorophyll contents of *D. salina* cells was obtained at 1% CO_2_. The chlorophyll contents of *D. salina* cells under 30% CO_2_ was much higher than these of incubation in air. The above results were consistent with cell biomass. During photosynthesis, oxygen and light generate reactive oxygen species (ROS). As a defense mechanism, organisms produce many endogenous antioxidants to eliminate harmful ROS, thus maintaining normal cellular function and organismal health (Ascherio and Schwarzschild, 2017). Carotenoidscan protect chlorophyll from photooxidation by absorbing thermal energy from singlet oxygen and quenching it by releasing energy through polyene vibrations (Abreu et al., 2020). As shown in Figure 1 F, the contents of carotenoid in *D. salina* cells cultivated with 20%–30% CO_2_ were higher than that in *D. salina* cells cultivated with 0.30%–10% CO_2_. This indicates that *D. salina* cells were stressed by ROS under 20%–30% CO_2_ conditions and produced carotenoids as antioxidants to eliminate the damage caused by ROS.

### 3.2 Effect of different CO_2_ treatments on overall transcriptional changes

**FIGURE 2:**
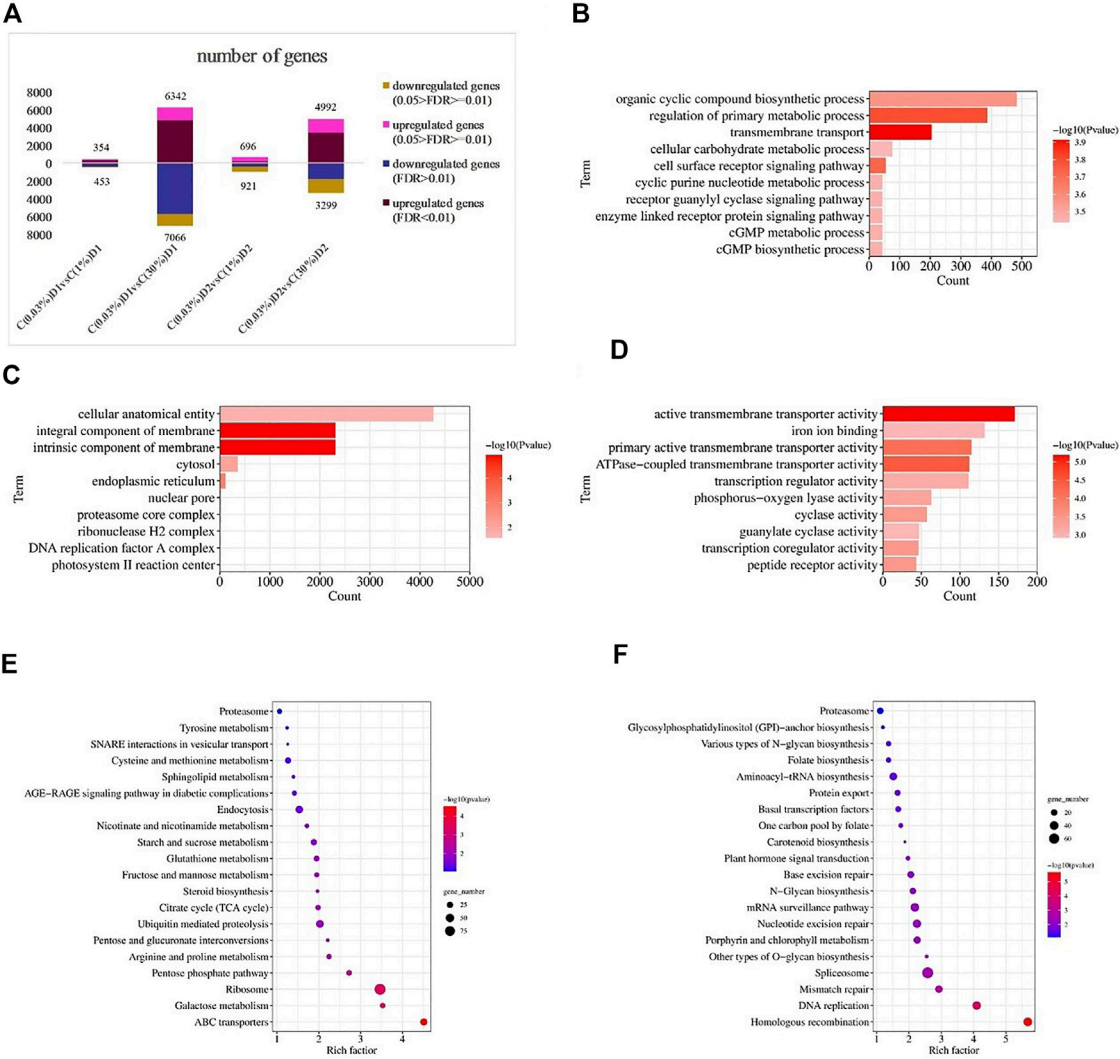
Transcriptional responses of *D. salina* to high CO_2_ stress. Number of differentially expressed genes (DEGs) between different CO_2_ treatment groups **(A)**. Distribution of top GO categories enrichment under 0.03% and 30% CO2 treatment (1 day): **(B)** (biological processes), **(C)** (cellular components) and **(D)** (molecular functions). Distribution of top KEGG metabolic pathways enrichment under 0.03% and 30% CO_2_ treatment (1 day): **(E)** (up-regulated genes) and **(F)** (down-regulated genes). Enrichment was analyzed based on hypergeometric test and Bonferroni adjustment (corrected *p*-value (*P*-adjust) < 0.05).

Twelve samples were used for transcriptome assembly, and a total of 97.67 Gb Clean Data were obtained. The Clean Data of each sample was more than 7.28 Gb, and the percentage of Q30 base was more than 95.06%. Statistical analysis of raw counts was performed by DESeq2 software based on negative binomial distribution, and genes with expression differences between comparison groups were obtained basing on certain screening conditions (p-adjust<0.05 and |log_2_FC|≥ 1). Compared with C (0.03%)D1, there were 807 differential genes in C (1%)D1 group, including 354 up-regulated genes and 453 down-regulated genes. Compared with C (0.03%)D1, C (30%)D1 group has 13408 differential genes, including 6342 up-regulated genes and 7066 down-regulated genes (Figure 2A).

To further elucidate the response of *D. salina* under high CO_2_ conditions, DEGs between C(0.03%)D1 and C(30%)D1 were subjected to GO analysis using the software Goatools (P-adjust<0.05). A total of 6039 DEGs were annotated in GO and divided into 225 functional subcategories, containing 137 groups of biological processes, 28 groups of cellular components and 60 groups of molecular functions. Within the category of biological processes, transmembrane transport, regulation of primary metabolic process, cell surface receptor signaling pathway, organic cyclic compound biosynthetic process, and cGMP biosynthetic process were prominent (Figure 2B). Within the category of cellular components, intrinsic component of membrane, integral component of membrane, endoplasmic reticulum, cytosol, and proteasome core complex were significantly enriched (Figure 2C). For the molecular functional categories, active transmembrane transporter activity, ATPase-coupled transmembrane transporter activity, primary active transmembrane transporter activity, transcription coregulator activity, peptide receptor activity were significantly enriched (Figure 2D).

In addition, KEGG enrichment analysis showed that 4056 DEGs were allocated to 125 KEGG pathways. The up-regulated genes (Figure 2E) are mainly enriched in ABC transporters, ribosome, galactose metabolism, pentose phosphate pathway, pentose and gluconate interconversions. The down-regulated genes (Figure 2F) were mainly enriched in homologous recombination, DNA replication, mismatch repair, other types of O-glycan biosynthesis, spliceosome, etc.

### 3.3 Differentially expressed genes associated with major metabolic pathways

#### 3.3.1 Differentially expressed genes related to photosynthesis and light protection

**FIGURE 3:**
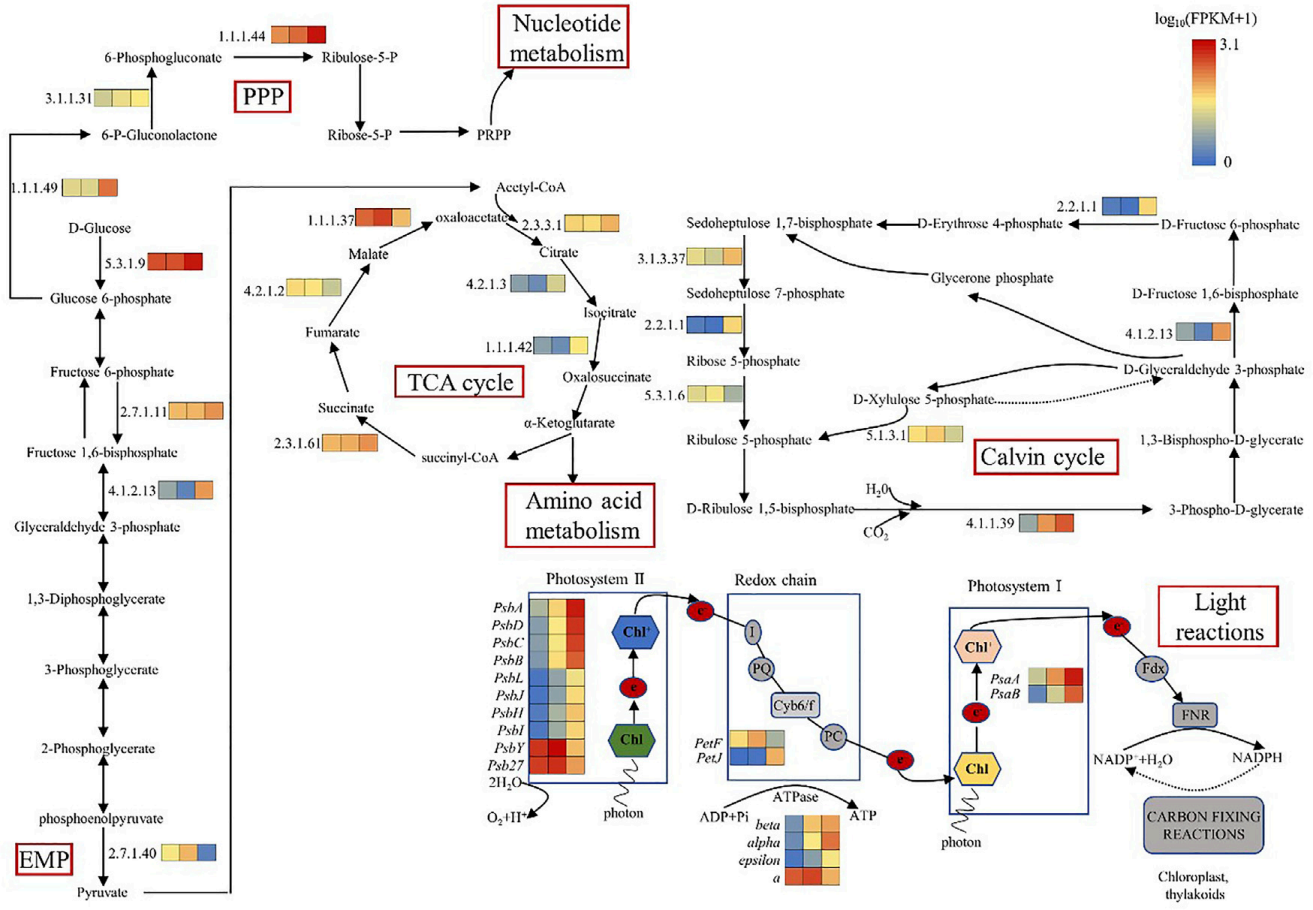
Changes in transcription levels of key enzymes in major metabolic pathways.

In the oxygen releasing complex, the electrons generated by splitting water molecules successively pass through PSII, Cytb6f and PSI, and finally pass to nicotinamide adenine dinucleotide phosphate (NADP) to form NADPH. The transmembrane proton gradient generated in this process drives ATP synthase to form ATP (Iverson, 2006). The core component of PSII core complex is D1 (*PsbA*)/D2 (*PsbD*) heterodimer, which can bind the central chlorophyll molecule and the primary electron acceptor and primary electron donor in the electron transfer process after photochemical reaction. The core antennas CP47 and CP43 of the optical system II (PSII) are combined at the side of the D1 (*PsbA*)/D2 (*PsbD*) core. CP47 and CP43 can not only function as core antennas, but also play an important role in maintaining the core structure of the PSII (Minagawa and Takahashi, 2004). *PsaA* and *PsaB* form a central heterodimer, which contains the components of reaction center P700 and electron transport chain (ETC), A_0_, A_1_ and F_X_ (Busch and Hippler, 2011). The expression levels of genes encoding D1 (*PsbA*), cp47 (*PsbB*), cp43 (*PsbC*), D2 (*PsbD*), *PsaA*, and *PsaB* were up-regulated in C(30%) D1 compared with C(0.03%) D1(Table 1). Cytb6f complex, containing subunits of Cytb6, Cytf and Fe-S protein, is responsible for transferring electrons from PQ to PC and converting light energy into transmembrane proton gradients for ATP synthesis (Mirkovic et al., 2017). The expression levels of cytochrome b6 (*PetB*), cytochrome b6-f complex subunit 4 (*PetD*), apocytochrome f (*PetA*) and cytochrome b6-f complex iron sulfur subunit (*PetC*) in the cytochrome b6f complex were significantly up-regulated in C (30%) D1 compared with C (0.03%) D1. Compared with C (0.03%) D1, the gene expression level of F type ATPase coding subunit(α,β,ε and a) were up-regulated in C(30%)D1 (Figure 3). It showed that under the condition of high concentration of CO_2_, *D. salina* had a strong response in the stage of photosynthesis and light reaction in a short time, and its demand for energy ATP increased.

**TABLE 1:** Differential expression of key proteins in photosynthesis

EC number/gene name	gene ID	Annotation	C(0.03%)D1 VS C(30%)D1
PsbA	TRINITY_DN1049_c0_g4	photosystem II P680 reaction center D1 protein	7.376
PsbD	TRINITY_DN3012_c0_g1	photosystem II P680 reaction center D2 protein	8.152
PsbC	TRINITY_DN1000_c0_g1	photosystem II CP43 chlorophyll apoprotein	7.978
PsbB	TRINITY_DN1134_c1_g1	photosystem II CP47 chlorophyll apoprotein	7.421
PsbL	TRINITY_DN8780_c0_g2	photosystem II PsbL protein	7.368
PsbJ	TRINITY_DN4268_c0_g1	photosystem II PsbJ protein	8.379
PsbH	TRINITY_DN35600_c0_g2	photosystem II PsbH protein	7.697
PsbI	TRINITY_DN6624_c1_g2	photosystem II PsbI protein	7.689
PsbY	TRINITY_DN4460_c0_g2	photosystem II PsbY protein	−2.611
Psb27	TRINITY_DN1881_c0_g1	photosystem II Psb27 protein	−1.663
PsaA	TRINITY_DN198_c0_g1	photosystem I P700 chlorophyll a apoprotein A1	6.811
PsaB	TRINITY_DN1934_c0_g1	photosystem I P700 chlorophyll a apoprotein A2	8.744
PetB	TRINITY_DN44490_c0_g1	cytochrome b6	6.776
PetD	TRINITY_DN17347_c0_g1	cytochrome b6-f complex subunit 4	7.007
PetA	TRINITY_DN1049_c0_g3	apocytochrome f	5.250
PetC	TRINITY_DN1498_c0_g1	cytochrome b6-f complex iron-sulfur subunit	10.422
PetF	TRINITY_DN1184_c0_g1	ferredoxin	−2.384
PetJ	TRINITY_DN42148_c0_g1	cytochrome c6	12.845
Beta	TRINITY_DN40385_c0_g1	F-type H+/Na + -transporting ATPase subunit beta	6.375
Alpha	TRINITY_DN6624_c0_g1	F-type H+/Na + -transporting ATPase subunit alpha	7.718
Epsilon	TRINITY_DN1049_c0_g5	F-type H + -transporting ATPase subunit epsilon	7.249
A	TRINITY_DN3078_c0_g1	F-type H + -transporting ATPase subunit a	−1.498
7.1.1.6	TRINITY_DN1498_c0_g1	cytochrome b6-f complex iron-sulfur subunit	10.422
4.1.1.39	TRINITY_DN39350_c0_g1	ribulose-bisphosphate carboxylase large chain	6.600
2.2.1.1	TRINITY_DN2780_c0_g1	transketolase	6.830
3.1.3.37	TRINITY_DN10394_c0_g2	sedoheptulose-bisphosphatase	2.177
5.3.1.6	TRINITY_DN682_c1_g2	ribose 5-phosphate isomerase A	−1.276
5.1.3.1	TRINITY_DN8089_c0_g2	ribulose-phosphate 3-epimerase	−1.092
4.1.1.31	TRINITY_DN1363_c2_g2	phosphoenolpyruvate carboxylase	−1.270
2.6.1.1	TRINITY_DN12116_c0_g1	aspartate aminotransferase, cytoplasmic	1.372
4.1.1.49	TRINITY_DN2184_c0_g1	phosphoenolpyruvate carboxykinase (ATP)	4.828
2.6.1.2	TRINITY_DN991_c0_g4	glutamate—glyoxylate aminotransferase	−6.366
1.1.1.39	TRINITY_DN14011_c0_g7	ATP-dependent RNA helicase DDX35	−1.552
1.1.1.40	TRINITY_DN1866_c0_g3	malate dehydrogenase (oxaloacetate-decarboxylating) (NADP+)	−1.424

#### 3.3.2 Differential expression genes related to carbon fixation pathway

Ribulose 1,5-diphosphate carboxylase (Rubisco enzyme) is a key enzyme in the process of carbon fixation in the Calvin cycle, which catalyzes CO_2_ to produce organic sugars (Parikh et al., 2006). As shown in Table 1 and 2, compared with C(0.03%)D1, the genes encoding ribulose diphosphate carboxylase (4.1.1.39), fructose diphosphate aldolase (4.1.2.13), ketotransferase (2.2.1.1) and Sedum heptanulose diphosphatase (3.1.3.37) in C(30%)D1 were up-regulated. However, the genes encoding ribose 5-phosphate isomerase (5.3.1.6) and ribulose phosphate 3-epimerase (5.1.3.1) in C (30%)D1 were down-regulated. These results indicated that the increased carbon fixation rate of *D. salina* under high CO_2_ conditions could be attributed to the following two factors: on the one hand, competition for Rubisco enzyme binding sites in the chloroplast matrix was intensified by CO_2_. On the other hand, the increase of CO_2_ concentration inhibited the photorespiration of algae to a certain extent and thus improve its net photosynthetic efficiency (Barati et al., 2021; Kselíková et al., 2022).

**TABLE 2:** Differential expression of key proteins in central carbon metabolism.

EC number/gene name	gene ID	Annotation	C(0.03%)D1 VS C(30%)D1
2.7.1.1	TRINITY_DN1846_c0_g1	hexokinase	−1.662
5.1.3.3	TRINITY_DN4105_c0_g5	aldose 1-epimerase	−1.427
5.1.3.15	TRINITY_DN8162_c0_g2	glucose-6-phosphate 1-epimerase	−1.699
5.3.1.9	TRINITY_DN13923_c0_g2	glucose-6-phosphate isomerase	1.701
2.7.1.11	TRINITY_DN6661_c0_g2	6-phosphofructokinase 1	1.325
4.1.2.13	TRINITY_DN2924_c0_g1	fructose-bisphosphate aldolase, class I	5.342
3.1.3.80	TRINITY_DN1745_c1_g1	2,3-bisphosphoglycerate 3-phosphatase	−1.402
4.1.1.49	TRINITY_DN2184_c0_g1	phosphoenolpyruvate carboxykinase (ATP)	4.828
2.7.1.40	TRINITY_DN6188_c0_g1	pyruvate kinase	−5.091
1.2.4.1	TRINITY_DN17294_c0_g2	pyruvate dehydrogenase E1 component alpha subunit	−1.885
4.1.1.1	TRINITY_DN13210_c0_g2	pyruvate decarboxylase	1.943
2.3.1.12	TRINITY_DN2835_c0_g1	pyruvate dehydrogenase E2 component	−1.050
1.8.1.4	TRINITY_DN3877_c0_g2	dihydrolipoamide dehydrogenase	−2.544
1.2.1.3	TRINITY_DN4984_c0_g1	aldehyde dehydrogenase family 7 member A1	4.890
4.1.1.49	TRINITY_DN2184_c0_g1	phosphoenolpyruvate carboxykinase (ATP)	4.828
2.3.1.12	TRINITY_DN2835_c0_g1	pyruvate dehydrogenase E2 component	−1.050
1.2.4.1	TRINITY_DN17294_c0_g2	pyruvate dehydrogenase E1 component alpha subunit	−1.885
1.8.1.4	TRINITY_DN3877_c0_g2	dihydrolipoamide dehydrogenase	−2.544
2.3.3.1	TRINITY_DN4322_c0_g1	citrate synthase	1.169
4.2.1.3	TRINITY_DN2602_c0_g1	aconitate hydratase	2.735
1.1.1.42	TRINITY_DN3276_c0_g1	isocitrate dehydrogenase	3.900
2.3.1.61	TRINITY_DN3065_c0_g3	dihydrolipoamide succinyltransferase	1.309
1.8.1.4	TRINITY_DN3877_c0_g2	dihydrolipoamide dehydrogenase	−2.544
6.2.1.4	TRINITY_DN860_c0_g2	succinyl-CoA synthetase beta subunit	−1.407
4.2.1.2	TRINITY_DN5127_c0_g3	fumarate hydratase, class I	−1.470
1.1.1.37	TRINITY_DN3047_c0_g1	malate dehydrogenase	−1.365
1.1.1.49	TRINITY_DN2433_c0_g1	glucose-6-phosphate 1-dehydrogenase	3.005
3.1.1.31	TRINITY_DN5229_c0_g2	6-phosphogluconolactonase	2.410
1.1.1.44	TRINITY_DN262_c0_g1	6-phosphogluconate dehydrogenase	1.712

#### 3.3.3 Differential expression genes related to central carbon metabolism

Glycolysis and gluconeogenesis share most reversible enzymes. However, they use different enzymes in the key steps (Lv et al., 2019). As shown in Table 2; Figure 3, compared with C (0.03%) D1, the gene expression levels of hexokinase (5.3.1.9), phosphofructokinase (2.7.1.11) and fructose diphosphate aldolase (4.1.2.13) encoding the key enzymes of glycolysis pathway in C (30%) D1 were significantly up-regulated. In addition, gene expression levels of some key enzymes involved in TCA cycle in C (30%)D1, including citrate synthase (2.3.3.1), aconitase (4.2.1.3), isocitrate dehydrogenase (1.1.1.42) and succinyl-CoA synthase (2.3.1.61), were significantly up-regulated. However, gene expression levels of fumarase (4.2.1.2) and malate dehydrogenase (1.1.1.37), key enzymes encoding TCA cycle pathway, were significantly down-regulated. The results showed that the metabolic rate of *D. salina* increased and the expression levels of fumarase and malate dehydrogenase decreased under the condition of short time exposure to high CO_2_, which may be due to the intermediate products in TCA cycle entering the amino acid metabolism and other pathways. Pentose phosphate pathway provides NADPH for biosynthesis, and its reversible non oxidized part is also an important source of carbon skeleton for the synthesis of nucleotides, aromatic amino acids, phenylpropanes and their derivatives. The key enzymes of pentose phosphate pathway are 6-phosphate glucose dehydrogenase and 6-phosphate gluconate dehydrogenase, which are involved in the pentose phosphate pathway to produce NADPH and ribonuclease 5-phosphate (Ru-5-P). Compared with C (0.03%) D1, we found that the gene expression levels encoding phosphogluconate dehydrogenase (1.1.1.49), phosphogluconate lactone (3.1.1.31) and phosphogluconate dehydrogenase (1.1.1.44) in C (30%) D1 were significantly up-regulated (Table 2). It shows that *D. salina* can produce more NADPH for biosynthesis and provide raw materials for the synthesis of other carbon skeletons when exposed to high CO_2_ for a short time.

### 3.4 Differential expression genes related to oxidative stress

As mentioned above the low pH is associated with high CO_2_ concentrations, this could induce serious oxidative stress and lead to ROS production in microalgal cells (Thompson et al., 2017). As the main executor of ROS scavenging system, the antioxidant system of algae consists of enzymatic antioxidant system and non-enzymatic antioxidant system. Their main function is to scavenge active oxygen (Liao et al., 2018). Enzymatic antioxidant system includes various antioxidant enzymes, such as superoxide dismutase (SOD), catalase (CAT), thioredoxin peroxidase (TrxR), ascorbic acid peroxidase (APX), glutathione peroxidase (GPX). Non-enzymatic antioxidant system includes various antioxidants, such as ascorbic acid (vitamin C), glutathione, vitamin E, carotenoids and proline (Serrano et al., 2021). SOD is the first line of defense in the enzymatic active oxygen scavenging pathway of algae. It is a metal enzyme family that can catalyze the transformation of superoxide into oxygen and hydrogen peroxide (H_2_O_2_) through disproportionation reaction (Zelko et al., 2002; Ben Hamouda et al., 2022). CAT is a tetramer enzyme containing heme, which is found in all aerobic organisms. CAT mainly exists in peroxides. Because its activity does not require reductive substrates and its high maximum reaction rate value and low Michaelis constant value to H_2_O_2_, it is essential to eliminate H_2_O_2_ produced in large quantities when cells are under stress (Gauthier et al., 2020). APX is found in higher plants, eukaryotic algae and some cyanobacteria (Roy et al., 2021). It exists in a variety of organelles, such as chloroplasts, mitochondria, peroxides and cytoplasm, and is the key enzyme to remove H_2_O_2_ in chloroplasts. Many studies have shown that stress has a significant impact on the activity of the antioxidant enzyme system of algae, but there are differences in the response of various enzymes to different stresses in different algal strains (Gauthier et al., 2020). Besides, the HSP/chaperone network includes Hsp70 family genes (*DnaK*, *DnaJ*, *DnaJC7*, *DnaJC11*, *DnaJC13*, *Hsp70*), Hsp family D gene (*HspD1*) and small Hsp genes (*Hsp20*, *Hsp33*) (Jacob et al., 2017). Compared with C(0.03%)D1, we found that the expression levels of genes encoding SOD, CAT and APX were significantly up-regulated in C(30%)D1. Compared to C(0.03%) D1, we found that the expression levels of genes encoding SOD, catalase CAT and APX were significantly up-regulated in C (30%) D1. The expression levels of genes encoding *DnaK*, *DnaJ*, *Hsp20*, *HspA5* and *HspA4* were significantly up-regulated in C (30%) D1(Table 3). The high expression of heat shock proteins indicated that *D. salina* was subjected to oxidative stress. On the other hand, *D. salin*a can respond to high CO_2_ stress through the antioxidant enzyme system under high CO_2_ stress.

**TABLE 3:** Differential expression of key proteins in oxidative stress.

EC number/gene name	gene ID	Annotation	C(0.03%)D1 VS C(30%)D1
CAT	TRINITY_DN7205_c0_g1	catalase	2.283
SOD1	TRINITY_DN12851_c0_g1	superoxide dismutase, Cu-Zn family	3.169
SOD2	TRINITY_DN26325_c0_g3	superoxide dismutase, Fe-Mn family	5.077
E1.11.1.11	TRINITY_DN14_c0_g2	l-ascorbate peroxidase	4.280
DnaK	TRINITY_DN3331_c0_g1	molecular chaperone DnaK	3.547
DnaJ	TRINITY_DN19711_c0_g1	molecular chaperone DnaJ	4.138
HspA5	TRINITY_DN4584_c0_g1	endoplasmic reticulum chaperone BiP	3.450
HSP20	TRINITY_DN2374_c1_g1	HSP20 family protein	9.021
HSPA4	TRINITY_DN8187_c0_g1	heat shock 70 kDa protein 4	4.479

## 4 Conclusion

In this study, one marine algal strain *D. salina* was cultured under 0.03%-30 % CO_2_ and 3% NaCl conditions. Results showed that *D. salina* produced a maximum biomass of 1.7 g·L^−1^ at 1 % CO_2_ and a biomass of 1.13 g·L^−1^ at 30% CO_2_, which was 2.79 and 1.85 times higher than that of incubation in air (0.03%). Under high CO_2_ stress, *D. salina* can eliminate ROS by synthesizing endogenous antioxidant carotenoids. In addition, the results of transcriptomic analysis also indicated that some key genes related to enzymatic antioxidant system and non-enzymatic antioxidant system antioxidant enzymes were up-regulated. Meanwhile, *D. salina* responded to high CO_2_ stress by promoting central carbon metabolism to produce more energy and enhancing photosynthesis to promote carbon fixation.

## Data Availability

The data presented in the study are deposited in the NCBI Sequence Read Archive repository, accession number PRJNA901516.
